# Assistive Technology to Support Dementia Management: A Scoping Review of Reviews

**DOI:** 10.1002/alz70858_102449

**Published:** 2025-12-24

**Authors:** Chaitali Desai, Erica Dove, Alyssa Benitez, Jarshini Nanthakumar, Emilia Main, Heather Colquhoun, Arlene Astell, Alex Mihailidis, Natasha Layton, Amer M. Burhan, Brian C. Chan, Rosalie H. Wang

**Affiliations:** ^1^ University of Toronto, Toronto, ON, Canada; ^2^ KITE Research Institute, Toronto Rehabilitation Institute, University Health Network, Toronto, ON, Canada; ^3^ University Health Network, Toronto, ON, Canada; ^4^ Northumbria University, Newcastle upon Tyne, United Kingdom; ^5^ KITE ‐ Toronto Rehabilitation Institute, University Health Network, Toronto, ON, Canada; ^6^ Monash University, Melbourne, VIC, Australia; ^7^ Rehabilitation, Ageing and Independent Living (RAIL) Research Centre, Frankston, VIC, Australia; ^8^ Ontario Shores Centre for Mental Health Sciences, Whitby, ON, Canada; ^9^ Toronto Dementia Research Alliance, Toronto, ON, Canada; ^10^ Institute of Health Economics, Edmonton, AB, Canada

## Abstract

**Background:**

More than 60% of Canadians living with dementia reside in their own homes, and over 25% rely on care partners (family members, friends) for daily activity assistance. Assistive technology (AT) helps to maintain health, social support, and autonomy. AT comprises assistive products and services which are required for safe and effective product use. Persons with dementia and care partners often require multiple AT types. AT for dementia management is rapidly developing with abundant scientific literature, presenting challenges when efficiently navigating and extracting insights for policy and personal decision‐making. The aim of this scoping review is to synthesize review‐level evidence on AT to support dementia management for persons with dementia and care partners living at home. The range of AT types and characteristics, outcomes and conclusions from review‐level evidence are examined. Knowledge gaps and areas for further investigation regarding use and access to AT are identified.

**Method:**

The Joanna Briggs Institute's framework for conducting scoping reviews and the PRISMA‐ScR guidelines were applied. Six electronic databases were searched. Selection criteria followed the PCC framework: Population (persons with dementia, care partners, health care professionals (e.g., therapists who recommend AT), Concept (AT), and Context (home and community settings). A data charting template guided data extraction, numerical summarization, and content analysis.

**Result:**

Out of 10,978 unique citations identified, 39 articles met the inclusion criteria. Preliminary results show that AT types ranged from virtual reality systems (e.g., immersive goggles, game‐based platforms) for motor and cognitive training, voice‐activated technologies (e.g., Amazon, Alexa) for reminders and speech assistance, wearable devices for fall detection, and smartphones for communication. Key AT characteristics include portability, ease of learning, and task‐specific adaptability. However, knowledge gaps in distribution, assessment, and use of AT, plus small sample sizes, inconsistent outcome measure use, and limited economic analyses, restrict definitive conclusions to support decisions for AT recommendations.

**Conclusion:**

This is the first scoping review of reviews on this topic, providing comprehensive insights into AT types, characteristics, uses, and access for dementia management. This review charts a path toward creating more impactful AT solutions for persons with dementia and their care partners in their own homes and community settings.

